# The effects of novel macrocyclic chelates on the targeting properties of the ^68^Ga-labeled Gastrin releasing peptide receptor antagonist RM2

**DOI:** 10.1186/s13550-023-01005-1

**Published:** 2023-06-07

**Authors:** Yinwen Wang, Hongmei Yuan, Sufan Tang, Yang Liu, Ping Cai, Nan Liu, Yue Chen, Zhijun Zhou

**Affiliations:** 1 The Department of Nuclear Medicine, Affiliated Hospital of Southwest Medical University, Jiangyang District, Luzhou, Sichuan China; 2 Nuclear Medicine and Molecular Imaging Key Laboratory of Sichuan Province, Jiangyang District, Luzhou, Sichuan China; 3 Department of Pharmaceutics, School of Pharmacy, Southwest Medical University, Jiangyang District, Luzhou, Sichuan China; 4 Institute of Nuclear Medicine, Southwest Medical University, Jiangyang District, Luzhou, Sichuan China; 5 Department of Nuclear Medicine, Sichuan Provincial People’s Hospital, University of Electronic Science and Technology of China, Chengdu, Sichuan China

**Keywords:** GRPr, Bombesin, RM2, Chelators, AAZTA^5^, DATA^5m^, Gallium-68

## Abstract

**Background:**

The gastrin-releasing peptide receptor (GRPr) is a molecular target for the visualization of prostate cancer. Bombesin (BN) analogs are short peptides with a high affinity for GRPr. RM2 is a bombesin-based antagonist. It has been demonstrated that RM2 have superior in vivo biodistribution and targeting properties than high-affinity receptor agonists. This study developed new RM2-like antagonists by introducing the novel bifunctional chelators AAZTA^5^ and DATA^5m^ to RM2.

**Results:**

The effects of different macrocyclic chelating groups on drug targeting properties and the possibility of preparing ^68^Ga-radiopharmaceuticals in a kit-based protocol were investigated using ^68^Ga-labeled entities. Both new RM2 variants were labelled with ^68^Ga^3+^ resulting in high yields, stability, and low molarity of the ligand. DATA^5m^-RM2 and AAZTA^5^-RM2 incorporated ^68^Ga^3+^ nearly quantitatively at room temperature within 3–5 min, and the labelling yield for ^68^Ga-DOTA-RM2 was approximately 10% under the same conditions. ^68^Ga-AAZTA^5^-RM2 showed stronger hydrophilicity according to partition coefficient. Although the maximal cellular uptake values of the three compounds were similar, ^68^Ga-AAZTA^5^-RM2 and ^68^Ga-DATA^5m^-RM2 peaked more rapidly. Biodistribution studies showed high and specific tumor uptake, with a maximum of 9.12 ± 0.81 percentage injected activity per gram of tissue (%ID/g) for ^68^Ga-DATA^5m^-RM2 and 7.82 ± 0.61%ID/g for ^68^Ga-AAZTA^5^-RM2 at 30 min after injection.

**Conclusions:**

The conditions for complexation of DATA^5m^-RM2 and AAZTA^5^-RM2 with gallium-68 are milder, faster and require less amount of precursors than DOTA-RM2. Chelators had an evident influence on the pharmacokinetics and targeting properties of ^68^Ga-X-RM2 derivatives. Positively charged ^68^Ga-DATA^5m^-RM2 provided a high tumor uptake, high image contrast and good capability of targeting GRPr.

**Supplementary Information:**

The online version contains supplementary material available at 10.1186/s13550-023-01005-1.

## Background

The overexpression of peptide receptors in various types of tumors is becoming more evident, leading to a growing interest in their potential application in cancer diagnosis or targeted therapy [1]. One prominent example is the gastrin-releasing peptide receptor (GRPr), which is predominantly expressed mainly in human tumors such as prostate [2, 3], breast [4, 5] and gastrointestinal stromal tumors [6] and small-cell lung cancer [7]. Due to the benefits of targeting tumors with good vascular permeability and rapid access, the use of peptide ligands for radionuclide targeting of GRPr has been extensively studied for both imaging and therapy purposes [8].

The GRPr, also referred as bombesin receptor subtype 2 (BB2), is part of the G protein-coupled family of bombesin receptor, which includes neuromedin B receptor (NMBR/BB1), and bombesin receptor subtypes 3/4 (BB3/BB4). Of the GRPr-targeting peptide ligands, bombesin and its derivatives have gained considerable attention due to their high affinity. As a result, radiolabeled analogs of these BN-like peptides could be used on the imaging and treatment of GRPr-expressing tumors [9]. BN-based antagonists have demonstrated superior in vivo performance compared to agonists, with improved biodistribution, targeting capabilities, and other attributes [10–14]. A small peptide called RM2, with the chelator DOTA coupled to D-Phe-Gln-Trp-Ala-Val-Gly-His-Sta-Leu-NH2 via the cationic spacer 4-amino-1-carboxymethyl-piperidine (referred to as DOTA-RM2 in this study), has shown to be one of the potent antagonists with excellent pharmacokinetic properties, indicating that gallium-68 labeled DOTA-RM2 may serve as a good candidate for positron emission tomography (PET) imaging [15]. Currently, GRPr imaging appears to be complementary to prostate-specific membrane antigen (PSMA) imaging in preclinical studies and preliminary human studies in patients with primary limited prostate cancer [16, 17]. Several studies have shown that in recurrent prostate cancer, RM2-PET imaging may be more beneficial in patients with negative findings on conventional imaging, compared to PSMA-PET [18–20]. In addition, RM2-like drugs expected to be used for treatment have also been extensively studied and reported in recent years [21, 22].

It is well known that the pharmacokinetics and targeting potency of small peptides can be altered by structural modifications. Chelating agents for linking radionuclides are essential for targeting-based polypeptide conjugates. However, the use of different macrocyclic chelators had a profound influence on the biodistribution profile of the radiolabeled conjugates [23, 24]. This is because chelating agents may affect specific interactions between drug molecules and receptors as well as off-target activity. It might be argued that the chelator changes the overall charges and even affects the local charges, lipophilicity, and preferred conformation of the radiolabeled peptide [25, 26]. Therefore, it is not surprising that biodistribution and targeting properties may be significantly affected, as shown for many short peptides [27, 28].

Herein, we investigated in vivo imaging performance based on the targeting polypeptide moiety of the GRPr antagonist RM2 and a new type of chelating group (AAZTA^5^, DATA^5m^) of the resulting radioligand. In general, acyclic chelators are considered to have the characteristics of fast radiolabeling kinetics. The downside is the poor stability of the formed complexes [29]. In contrast, macrocyclic chelator-metal complexes are particularly stable, but the formation of complexes often requires high temperatures and consumes a longer period. The hybrid chelator AAZTA and DATA, as hexadentate tribasic ligands, derived from perhydro-1,4-diazepine, combine the advantages of cyclic and non-cyclic chelators [30, 31]. The endocyclic and exocyclic amines in this hybrid structures provide three ligand units and can introduce three or four further donor units through the alkylation of these amines with carboxylic acids [32]. Studies have shown that such hybrid chelators have excellent properties for nucleophiles such as gallium, lutetium, scandium, and copper, and can complex rapidly and stably with the corresponding metals at room temperature [33–36]. Their highly efficient and robust labelling characteristics of AAZTA and DATA render them promising candidates for utilization in ^68^Ga-PET and the development of kit-type labelling. In recent years, some small molecules have been further developed as radioligands with fast labeling properties, such as TOC [37], PSMA [36] and FAP inhibitors [34, 35]. To the best of our knowledge, AAZTA^5^ and DATA^5m^ have not been applied to the GRPr-targeted peptide RM2, and investigation into the biological properties of its derivatives are even more limited. Therefore, evaluating these new radiotracers with AAZTA^5^ and DATA^5m^ as ligands will be informative and valuable.

To investigate the impact of macrocyclic chelators on RM2 peptide, ^68^Ga-AAZTA^5^-RM2, ^68^Ga-DATA^5m^-RM2, and reference radioligand ^68^Ga-DOTA-RM2 were synthesized. We hypothesized that these chelators might display different profiles in vitro and in vivo; thus, a radiotracer with optimized properties in imaging or biodistribution will likely be screened. The physicochemical properties and the binding specificity to PC-3 cells of these ^68^Ga-labeled conjugates were evaluated. In addition, the distribution and PET/CT imaging of ^68^Ga-X-RM2 (*X* = DOTA or DATA^5m^ or AAZTA^5^) in PC-3 transplanted BALB/c nu/nu mice were also compared.

## Results

### Peptide synthesis

**Fig. 1:**
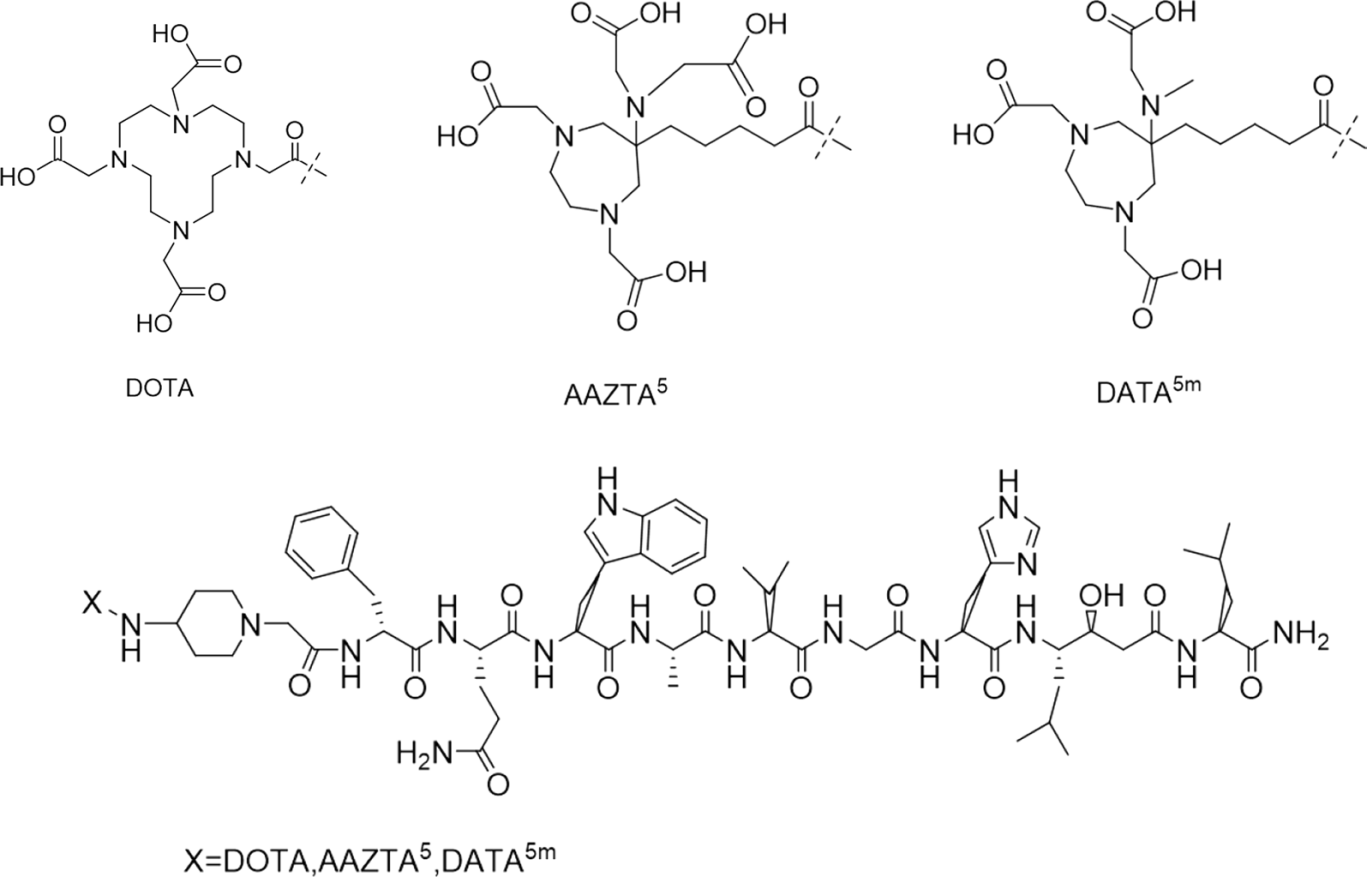
Chemical structures of chelators and peptide conjugates X-RM2

X-RM2 (Fig. 1) was synthesized using solid-phase peptide synthesis (Fmoc chemistry). The final products were analyzed by electrospray ionization-mass spectrometry (ESI–MS) and *m/z* values were provided in accordance with those expected. As determined with reversed-phase high-performance liquid chromatography (RP-HPLC) at UV_220_, the purity was more than 95% for all three peptides (Additional file 1: Fig. S1–S2; Table 1).

**Table 1:** Physical properties of X-RM2

Compound	Chemical formula	MW (g/mol)	*m*/*z*	Chemical purity (%)	Radiochemcial purity (%)^a^	Log *P*
DOTA-RM2	C_78_H_118_N_20_O_19_	1639.92	[M + H]^+^ 1640.80	> 95	98.00 ± 0.67	− 2.49 ± 0.17
DATA^5m^-RM2	C_79_H_119_N_19_O_19_	1638.94	[M + 2H]^2+^ 820.93	> 95	99.36 ± 0.58	− 2.29 ± 0.11
AAZTA^5^-RM2	C_80_H_119_N_19_O_21_	1682.94	[M − 2H]^2−^ 840.96	> 95	99.26 ± 0.90	− 2.69 ± 0.10

^a^The radiochemcial purity of ^68^Ga-X-RM2 were measured by RP-HPLC

### Radiolabeling, stability and LogP

**Fig. 2:**
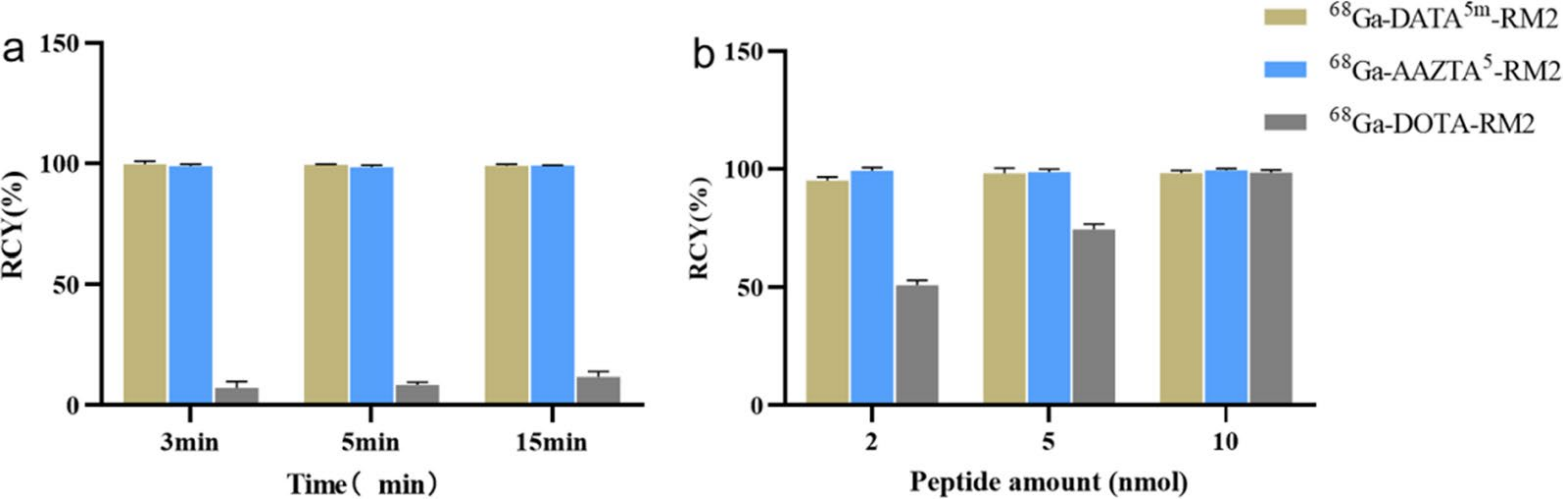
The system was maintained consistency in ^68^Ga^3+^ concentration, pH level, and solvent throughout the experimental groups. **a** Radiochemical yields of all compounds at room temperature at 3, 5, and 15 min. 10 nmol of the precursor was added to 1 ml of ^68^GaCl_3_ (approximately 150 MBq) and the pH was adjusted to 4.0–4.5 using the same volume of sodium acetate buffer solution. **b** Radiochemical yields of different amounts of precursors under suitable labeling conditions. Varying quantities of precursor were added to the same volume of ^68^GaCl_3_ (150 MBq) and sodium acetate solution. DATA^5m^- and AAZTA^5^-were reacted for 10 min at room temperature and DOTA-for the same time at 95 °C

The radiochemical purity of ^68^Ga-X-RM2 are > 95% (Additional file 1: Fig. S3). Compared with DOTA-RM2, AAZTA^5^-RM2 and DATA^5m^-RM2 had milder labelling conditions and much higher radiochemical yields at room temperature. AAZTA^5^-RM2 and DATA^5m^-RM2 incorporated ^68^Ga^3+^ nearly quantitatively (> 98%) at room temperature in 3 min, in contrast, the labelling yield of ^68^Ga-DOTA-RM2 was less than 10% under the same conditions or extended time (Fig. 2a). To explore the amount of precursors required, all compounds were subjected to optimal labelling conditions. The results showed that AAZTA^5^-RM2 and DATA^5m^-RM2 consumed only half of the precursors compared to DOTA-RM2 (Fig. 2b).

**Fig. 3:**
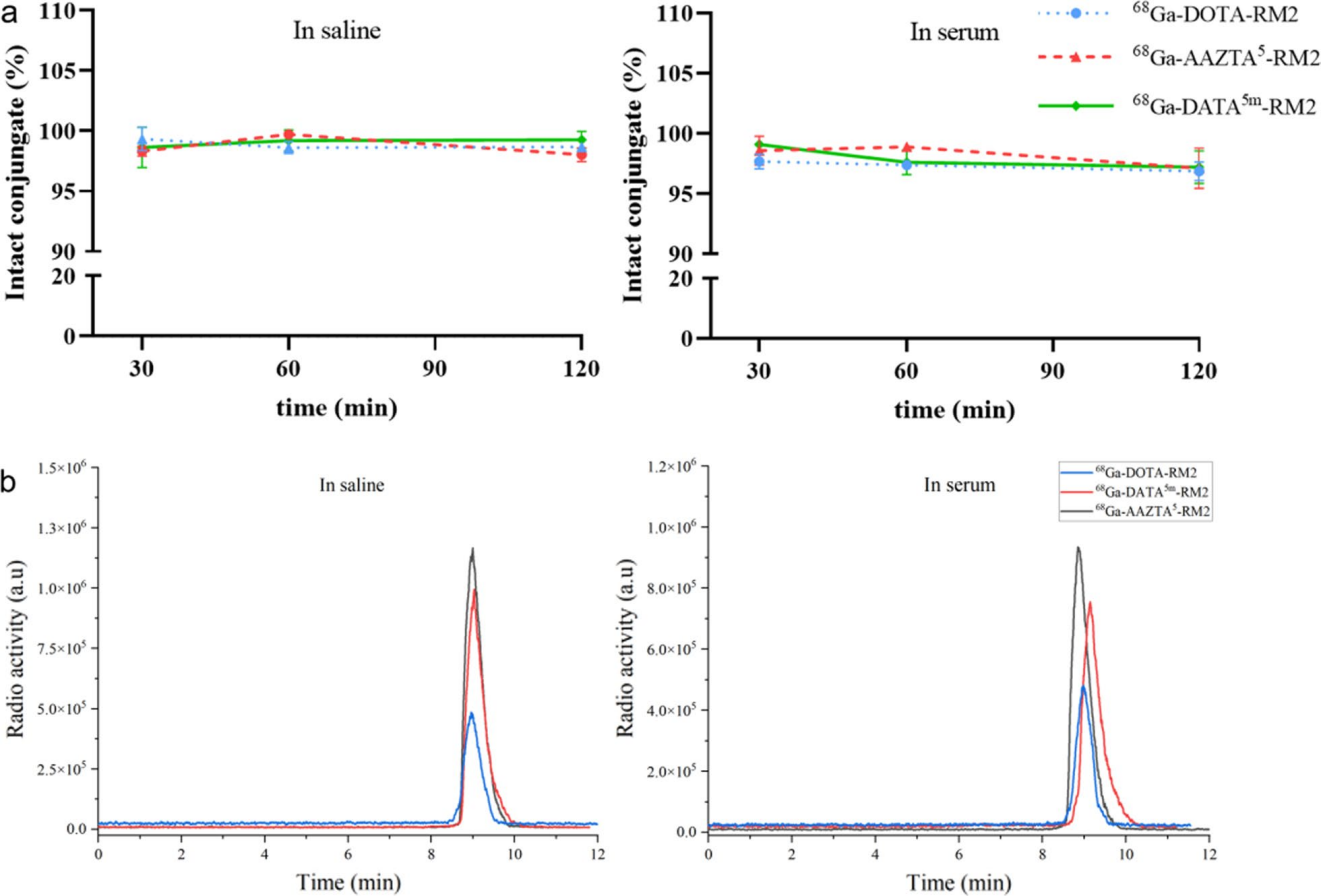
Stability of ^68^Ga-X-RM2. **a** Radiochemical purity was determined in saline and human serum at 30 min, 60 min, and 120 min. **b** RP-HPLC chromatograms of ^68^Ga-X-RM2 in saline and human serum at 120 min

In vitro stability studies were incubated in saline or human serum at 37 °C. Overall, all compounds showed excellent stability in the system at all time points (Fig. 3a). After 120 min, more than 95% of ^68^Ga-AAZTA^5^-RM2 and ^68^Ga-DATA^5m^-RM2 were stable in the system (Fig. 3b). As shown in Table 1, the LogP values of the three compounds are − 2.29 ± 0.11 (^68^Ga-DATA^5m^-RM2), − 2.69 ± 0.10 (^68^Ga-AAZTA^5^-RM2), and − 2.49 ± 0.17 (^68^Ga-RM2). The results showed that ^68^Ga-AAZTA^5^-RM2 was more hydrophilic than ^68^Ga-RM2 and ^68^Ga-DATA^5m^-RM2.

### Cell binding studies

**Fig. 4:**
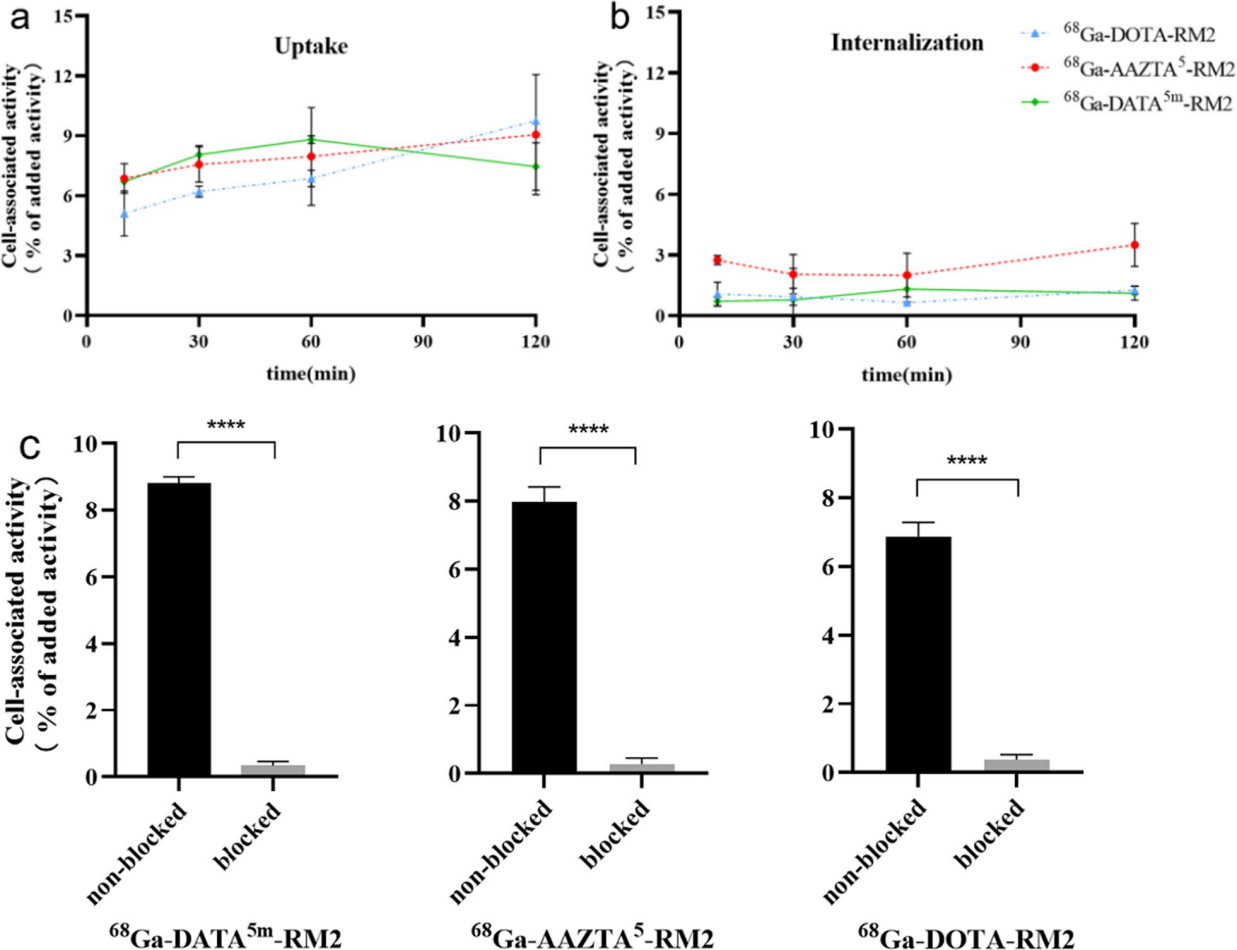
The uptake (**a**) and internalization (**b**) of ^68^Ga-X-RM2 in PC3 cells (normalized to 3 × 10^5^ cells) at 10 min, 30 min, 60 min, and 120 min. **c** In vitro binding specificity of ^68^Ga-X-RM2 tested on PC-3 cells at 60 min

The binding of ^68^Ga-X-RM2 to the PC-3 cells was rapid, and the cell-associated radioactivity reached a plateau within 2 h (Fig. 4a). The results showed the cellular uptake rate of ^68^Ga-AAZTA^5^- and ^68^Ga-DATA^5m^-was slightly higher than ^68^Ga-DOTA-RM2 within 1 h. However, the uptake of ^68^Ga-DATA^5m^-began to decrease after 1 h and reached a lower level compared to ^68^Ga-DOTA-RM2 after 2 h. Nonetheless, the overall cellular uptake rates of the three compounds did not differ significantly. Interestingly, the internalization value of ^68^Ga-AAZTA^5^-RM2 was significantly higher from the other two (Fig. 4b). To ensure that the GRPR-binding capacity and specificity of X-RM2 were preserved after labeling with ^68^Ga, an in vitro binding specificity test was performed on PC-3 cells. As shown in Fig. 4c, the results demonstrated that the binding of ^68^Ga-X-RM2 to the cells was receptor mediated because cell binding of radiolabeled compounds was significantly reduced after unlabeled DOTA-RM2 occupy the receptor (****p ≤ 0.0001).

### Imaging and biodistribution studies

**Fig. 5:**
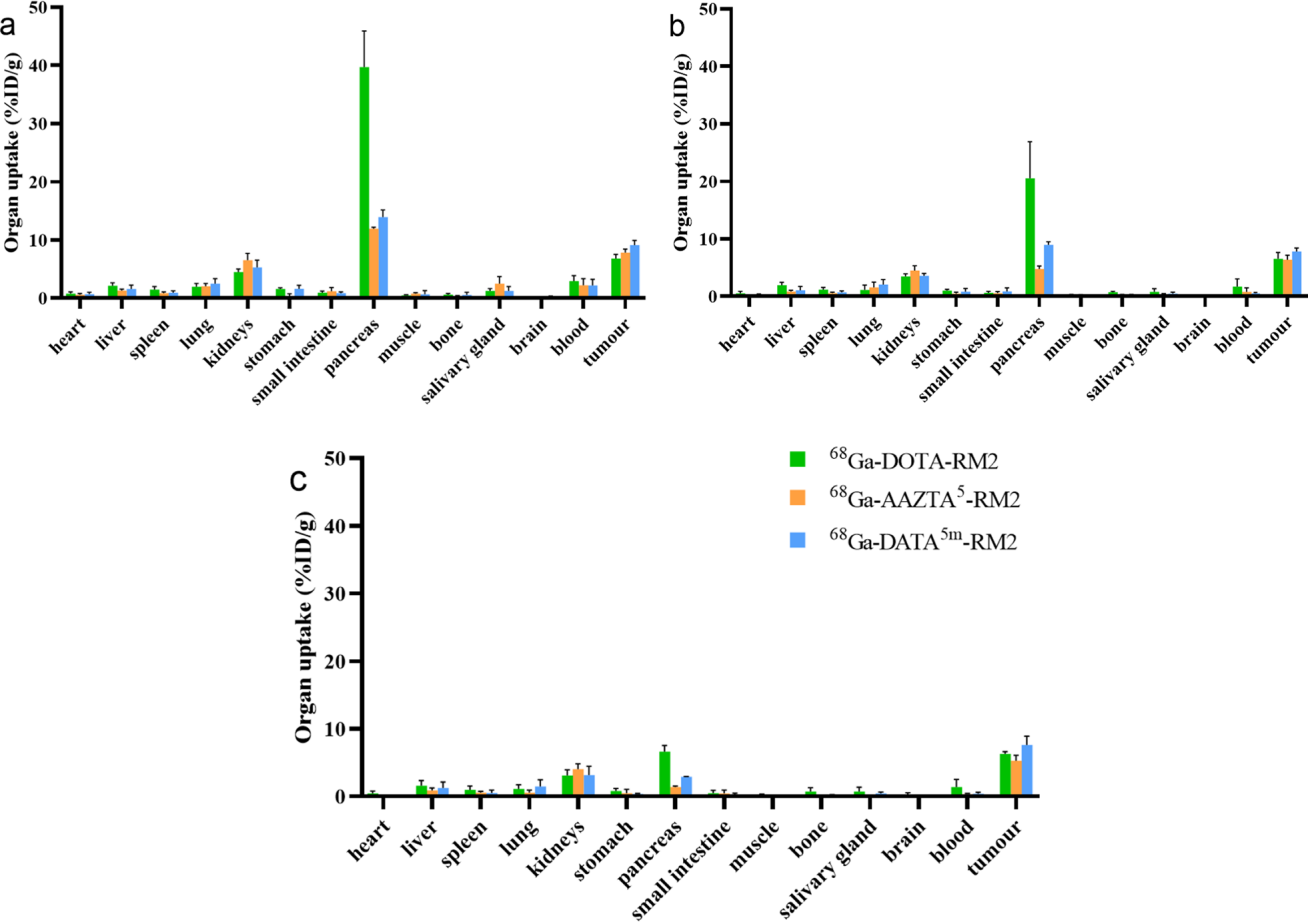
Organ biodistribution of ^68^Ga-X-RM2 expressed as % ID/g tissue at 30 min (**a**), 60 min (**b**), and 120 min (**c**) post-injection. Data are expressed as the means ± SD (*n* = 5)

The biodistribution of ^68^Ga-X-RM2 in BALB/C nu/nu mice bearing PC-3 xenografts at 30, 60, and 120 min were presented in Fig. 5 and Additional file 1: Table S1. Organ distribution with ^68^Ga-DATA^5m^-RM2 revealed high specific uptake in PC3 tumors and the maximum uptake value was 9.12 ± 0.81% ID/g after 30 min. In contrast, tumor uptake in ^68^Ga-AAZTA^5^-RM2 and ^68^Ga-DOTA-RM2 was lower than ^68^Ga-DATA^5m^-RM2 at any given time point. For normal organs, such as kidney, liver and muscle, the three compounds exhibited similar drug accumulation. We have noticed that the pancreatic uptake of these tracers is quite high, in particular with compound ^68^Ga-DOTA-RM2. After replacing DOTA with DATA^5m^ or AAZTA^5^, pancreatic uptake of ^68^Ga-DATA^5m^-RM2 and ^68^Ga-AAZTA^5^-RM2 was significantly reduced.

**Fig. 6:**
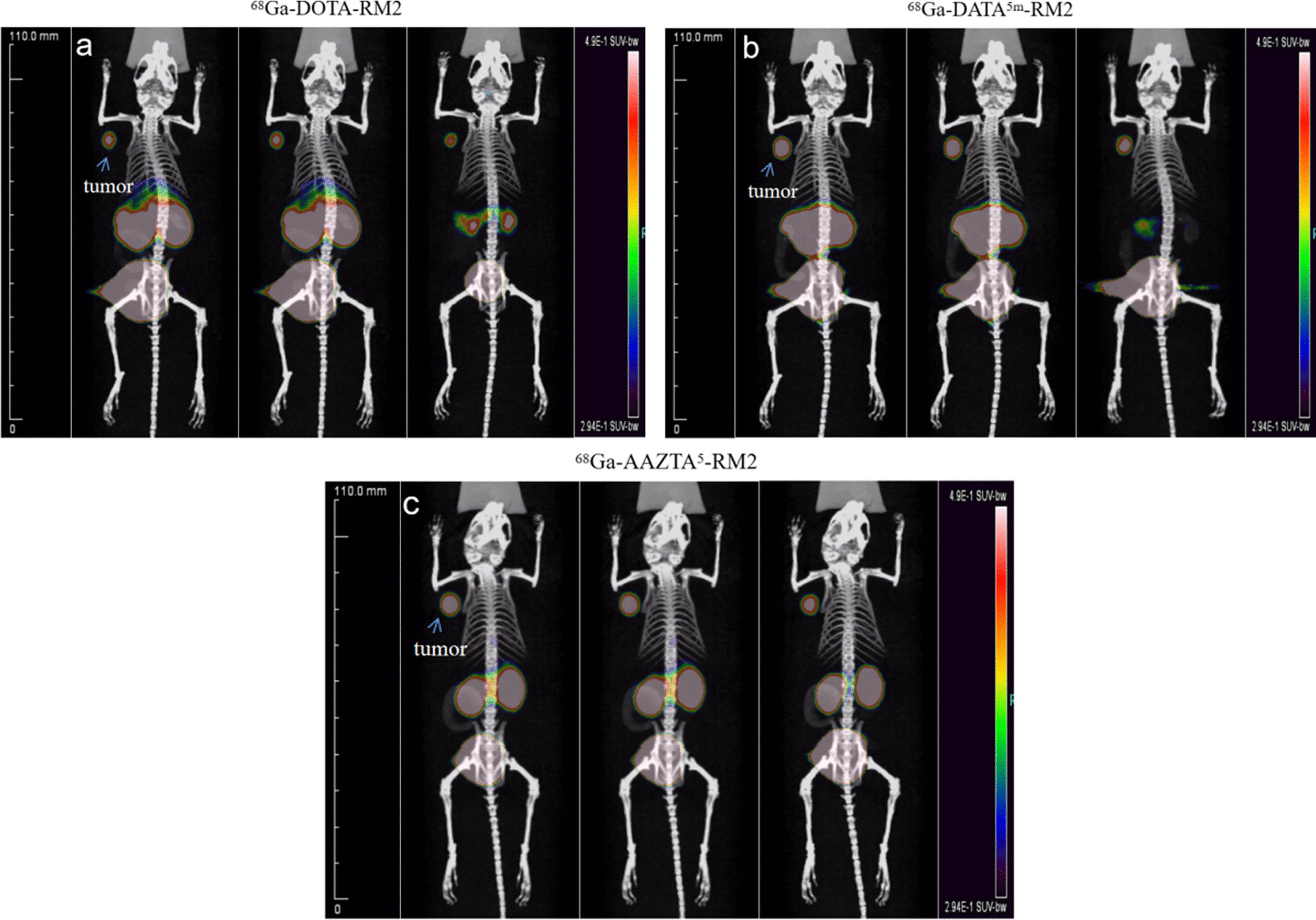
Maximum intensity projection of the whole-body coronal micro-PET/CT images of a BALB/c nu/nu male mouse bearing a PC3 tumor xenograft. Time-dependent static scans demonstrated the tumor-targeting efficacy of ^68^Ga-X-RM2 at 30, 60, and 120 min post-injection of ^68^Ga-DOTA-RM2 (**a**), ^68^Ga-DATA5m-RM2 (**b**) and ^68^Ga-AAZTA5-RM2 (**c**). Approximately 2.6 MBq/mice were injected

**Fig. 7:**
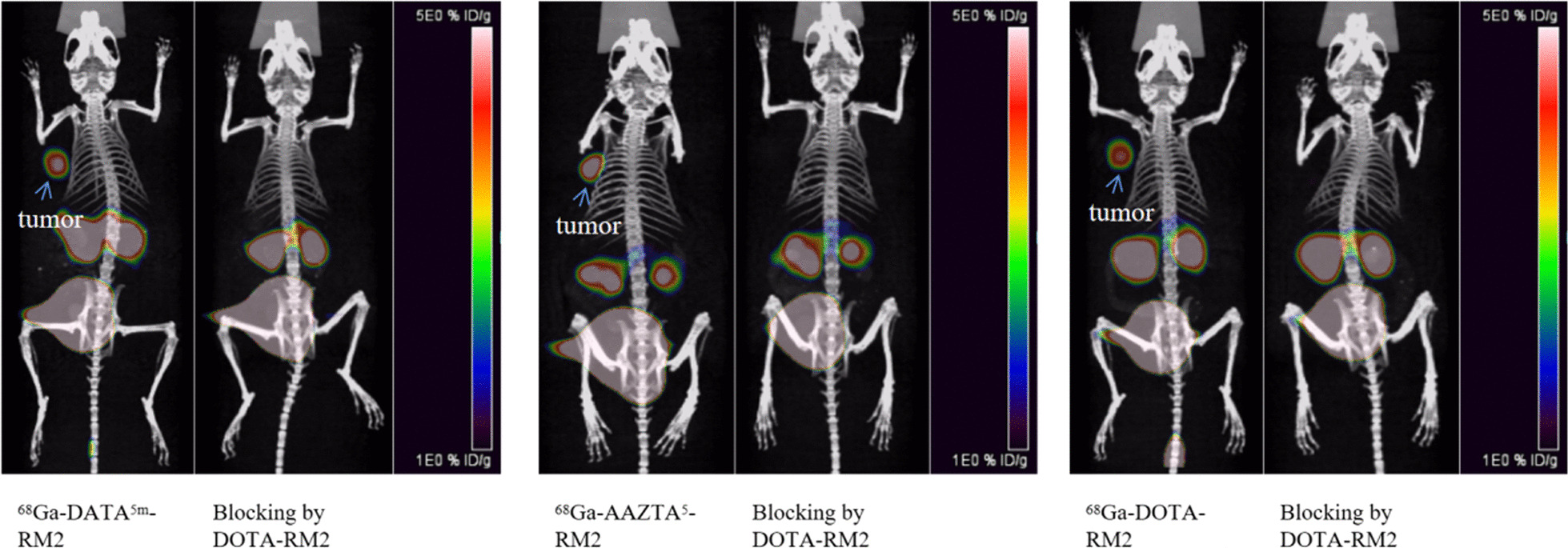
In vivo blocking and specificity of ^68^Ga-DATA^5m^-RM2, ^68^Ga-AAZTA^5^-RM2 and ^68^Ga-DOTA-RM2

Imaging. Subsequently, whole-body micro-PET/CT static imaging was performed on ^68^Ga-DATA5m-RM2 and ^68^Ga-AAZTA5-RM2 at 30, 60, and 120 min in PC-3 tumor-bearing mice (Fig. 6), using ^68^Ga-DOTA-RM2 as a reference. The results showed that ^68^Ga-DATA^5m^-RM2 and ^68^Ga-AAZTA^5^-RM2 had significant tumor uptake and even longer retention time than ^68^Ga-DOTA-RM2. Furthermore, quantitative data obtained from micro-PET/CT revealed that the mean %ID/g at the tumor for all three compounds reached its maximum within 30 min, while the rapid elimination of radioactivity was also observed in other organs, muscle, and blood, resulting in a clean background. The accumulation of all tracers in the renal collecting system and bladder was observed, reflecting the renal clearance of the drugs. As shown in Fig. 7, the uptake of ^68^Ga-DATA^5m^-RM2 and ^68^Ga-AAZTA^5^-RM2 was significantly reduced in tumors using unlabeled DOTA-RM2 as a blocking ligand. It indicates that the uptake of ^68^Ga-DATA^5m^-RM2 and ^68^Ga-AAZTA^5^-RM2 in tumors is mediated by GRPr consistent with ^68^Ga-DOTA-RM2.

## Discussion

An important role of chelators is to stably complex radionuclides and thus provide a means for labelling the targeting vector to allow the labelled ligands to interact with the targeted molecules. Many structure-related variables determine radiopharmaceuticals’ general behavior. In general, due to the relatively small size of the peptides, any structural modification of a substrate likely affects the affinity and selectivity to its targeted receptor. For example, the pharmacokinetics of peptides can be tuned by altering the chelators with variable lipophilicity, charge, size, and coordination symmetry [24]. In addition, the introduction of chelators may affect not only the binding but also the function of the targeted peptide, for example, by changing the agonistic effect to an antagonistic one [38]. Recently, the effects of organ distribution, target uptake and image contrast of RM2 derivatives with different chelating agents were investigated. The results show that the chelating agent largely affects the affinity of the molecule, which may be related to its total charge, with an improved affinity when the N-terminal carries an overall positive charge [39]. Thus, modification of chelating groups is one of the simple but effective ways to change the pharmacokinetic properties and targeting characteristics of peptides. This study aims to synthesize and compare RM2 analogs conjugated with homologous macrocyclic chelates and investigate the effect of these building blocks on the imaging performance.

Bifunctional AAZTA derivatives were recently reported and successfully applied to prepare ^68^Ga conjugates for PET imaging [30]. AAZTA^5^ is a suitable derivative that introduces a C5-alkyl spacer with a terminal carboxylic acid functional group on the AAZTA backbone. Incorporating the alkane chain may increase the lipophilicity of the molecule and on the other hand enrich its steric structure, such as chiral isomerism. The influence of the chirality and lipophilicity of a chelator on the biodistribution was estimated but have not been extensively investigated [26, 40]. Similarly, DATA^5m^ has the same backbone structure as AAZTA^5^, except that one of the carboxyl groups on the primary amine group is replaced by a methyl group. It may be facilitates the formation of stable coordination complexes with small metal ions and azacarboxylates based on 1,4,7-triazacyclononanes [41, 42]. Because the geometry of octahedra is favorable for the coordination of metal ions such as Zn^2+^, Mn^2+^, Fe^3+^ and Ga^3+^, and the presence of primary amine groups can easily lead to the nitrogen atom-functionalization of heptathlete ligands. In addition, the chelating portion in DATA^5m^ is hybrid because the scaffold contains both cyclic and acyclic structural features, where the flexibility of the acyclic portion facilitates fast complexation while the cyclic portion minimizes the energy barrier to complexation and inhibits the decomplexation processes [32]. This particular chemical property leads to a unique coordination mode with ^68^Ga [37].

The study replaced the chelator of DOTA-RM2 using AAZTA^5^ and DATA^5m^. Once labelled with ^68^Ga^3+^, the biological properties were investigated in vitro and in vivo. To ensure the comparability of the research, we performed a head-to-head comparative study along with DOTA-RM2. It turned out that lower temperatures, shorter times, and less quantities of precursors are required when labelling AAZTA^5^-RM2 and DATA^5m^-RM2 with ^68^Ga^3+^ (Fig. 2). The results showed that the radiolabeling of DATA^5m^-RM2 and AAZTA^5^-RM2 was achieved within 3 min at room temperature. This simplified the reaction conditions is advantageous over DOTA-RM2, which needs to be carried out at a higher temperature condition. At the same time, the amount of precursors required for the complexation of DATA^5m^ and AAZTA^5^ with ^68^Ga^3+^ was far less than that of DOTA, which was about 1/5–1/2 of that. In this context, a higher specific activity may be obtained for the same amount of DATA^5m^-RM2 or AAZTA^5^-RM2. Additionally, the simple and efficient labelling process might further facilitate the rapid kit preparation of ^68^Ga-radiopharmaceuticals, compared to previous kit-based protocols. The superior chemical properties of AAZTA^5^ and DATA^5m^ may enhance drug preparation efficiency, particularly for hospitals or institutions with inadequate supporting facilities.

Cellular uptake and internalization studies were conducted in PC3 cells to examine the impact of variations in chelating groups on receptor binding capacity. The results showed that all three compounds displayed highly specific binding to GRPr-expressing cells. While their maximal cellular uptake was similar, there was a slight downward trend in the uptake of ^68^Ga-DATA^5m^-RM2 after 2 h (Fig. 4a). In addition, it was observed that all three compounds were characterized by a low level of internalization, but AAZTA appeared to be slightly higher than the other ligands. (Fig. 4b). Although the reason was unclear, some studies had reported that the number of carboxylates of chelators may be an essential factor in determining the internalization rate [25]. In vitro and in vivo binding specificity assays showed that AAZTA^5^-RM2 and DATA^5m^-RM2 retained GRPr-binding capacity after ^68^Ga-labeling and confirmed that binding of both compounds to cells was receptor-mediated (Figs. 4c, 7). Thus, the introduction of these two chelating groups does not affect the specificity and targeting ability of the new ligand to bind to GRPr to a large extent. Indeed, DATA^5m^-RM2 and AAZTA^5^-RM2 are superior in radiochemistry properties, meanwhile, antagonistic property towards the receptor is remained.

Biodistribution studies were performed in PC-3 tumor-bearing mice to further explore the effect of chelators on targeting and other biological properties (Fig. 5; Additional file 1: Table S1). Consistent with the results of in vitro studies, ^68^Ga-DATA^5m^-RM2 and ^68^Ga-AAZTA^5^-RM2 showed similar biodistribution compared to ^68^Ga-DOTA-RM2. This was reflected in the similar drug accumulation in tumor and non-target organs for all three compounds. However, it was worth noting that the radioactivity accumulation in the pancreas (known as receptor-positive organs) was significantly lower for ^68^Ga-DATA^5m^-RM2 and ^68^Ga-AAZTA^5^-RM2 (Fig. 5). The pancreatic uptake of ^68^Ga-DATA^5m^-RM2 and ^68^Ga-AAZTA^5^-RM2 was only 1/3 or even lower than that of ^68^Ga-DOTA-RM2. The biodistribution of drugs can be influenced by multiple factors, with pharmacokinetics playing a significant role in determining drug accumulation in non-target organs. Our hypothesis is that the incorporation of a hybrid chelator into RM2 impacts its pharmacokinetic properties, as it may weaken the interactions within the microenvironment. This could result in a more rapid and reversible dissociation of RM2 from GRP receptors, thereby reducing its accumulation in the pancreas. In addition, tumor uptake was comparable for all tested analogs, but ^68^Ga-DATA^5m^-RM2 produced better tumor-to-organ ratios compared to ^68^Ga-DOTA-RM2, especially tumor-to-muscle/bone ratio (Table 2). Although the maximum tumor uptake of the ^68^Ga-AAZTA^5^-RM2 tended to decrease after 30 min, its tumor-to-organ ratios were still excellent. Taken together, compared with ^68^Ga-DOTA-RM2 and ^68^Ga-AAZTA^5^-RM2, ^68^Ga-DATA^5m^-RM2 showed better targeting effects and pharmacokinetic properties, which are favorable for imaging quality.

**Table 2:** Quantitative analysis of tumor-to-background ratios

Tumour/organ ratio	DATA^5m^-RM2			AAZTA^5^-RM2			DOTA-RM2		
	30 min	60 min	120 min	30 min	60 min	120 min	30 min	60 min	120 min
Tumour/liver	5.86	7.57	6.09	6.04	7.55	5.92	3.19	3.40	4.00
Tumour/kidneys	1.73	2.20	2.41	1.20	1.41	1.31	1.53	1.89	2.05
Tumour/muscle	25.42	56.83	62.23	10.33	37.63	60.70	14.41	21.80	31.73
Tumour/blood	4.23	14.56	18.21	3.53	8.38	15.64	2.35	3.88	4.58
Tumour/pancereas	0.65	0.87	2.60	0.66	1.33	3.78	0.17	0.32	0.96
Tumour/bone	16.17	29.29	50.21	21.84	36.04	57.53	11.16	8.99	8.60

Herein, we present the synthesis and in vitro and in vivo study of novel GRPr-targeted radiotracers, ^68^Ga-DATA^5m^-RM2 and ^68^Ga-AAZTA^5^-RM2. We further evaluated their affinity and specificity to GRPr-positive tumors by comparing the pharmacokinetics and PET imaging abilities with ^68^Ga-DOTA-RM2. ^68^Ga-DATA^5m^-RM2 and ^68^Ga-AAZTA^5^-RM2 displays favorable pharmacokinetics based on good tumor to non-target organs ratio. The excellent uptake and specificity in GRPr-positive tumors indicates that DATA^5m^-RM2 has no less targeting ability than DOTA-RM2. In addition, the DATA^5m^ chelators show improving radiolabeling characteristics, making them ideal candidates for developing a new generation of ^68^Ga-PET imaging agents that can be labelled in a kit-type manner. Therefore, the ^68^Ga-DATA^5m^-RM2 may be the preferable candidate for visualizing GRPr-expressing tumors using PET.

## Conclusion

This manuscript presents the in vitro and in vivo study of two new RM2 derivatives by introducing new chelators, DATA^5m^ and AAZTA^5^, into GRPr antagonist RM2. It shows that the chelator component affects the targeting properties and pharmacokinetics of the ^68^Ga-labeled BN antagonist RM2 to some extent. The results indicate that ^68^Ga-DATA^5m^-RM2 and ^68^Ga-DOTA-RM2 had comparable radioactive uptake in GRPr-positive tumors, but lower uptake in normal organs. Thus, ^68^Ga-DATA^5m^-RM2 offers a superior tumor-to-organ ratios, together with its much milder radiolabeling procedure, it might be a more viable candidate for further development as a PET agent for visualizing GRPr-positive tumors.

## Materials and methods

Reagents and solvents, which were purchased from commercial sources, were of analytical or HPLC grade. DATA^5m^-RM2, AAZTA^5^-RM2 and DOTA-RM2 were purchased from Nanchang Tanzhen Biological Technology Co., Ltd. (China). ^68^GaCl_3_ was eluted from a ^68^Ge–^68^Ga generator (ITM, Germany). Mass spectrometry (MS) was performed using an LC–MS system model LC-2030C (SHIMADZU, Japan). Chemical and radiochemical purity was determined by analytical high performance liquid chromatography (HPLC, SHIMADZU) equipped with a 4.6 mm × 250 mm C18 reversed-phase column (Agilent). All compounds were purified by preparative HPLC (Agilent) equipped with a 10 mm × 250 mm C18 reversed-phase column (Agilent). Radiocounting was performed using a CAPRAC-t γ-counter (Edmonton, Canada). Micro-PET/CT imaging using a small animal PET/SPECT/CT scanner, model InLiView-3000B (NOVEL MEDICAL, China). PC-3 cells were purchased from the Chinese Infrastructure of Cell Line Resource. BALB/c nude mice were purchased from Beijing HFK BIOSCIENCE Co., Ltd., China.

### Peptide synthesis

The peptide chain of RM2 was synthesized according to the previous method [43]. The peptide–chelator conjugate RM2 was synthesized manually according to standard Fmoc chemistry using Rink amide MBHA resin [23]. AAZTA^5^(^t^Bu)_3_ and DATA^5m^(^t^Bu)_4_ were also synthesized as previously reported [34, 35]. Then, the spacer and the chelator were consecutively coupled to the peptide with HATU as an activating agent.

### Radiolabeling and purification

20 µL (aqueous solution of 15 nmol) of X-RM2 was buffered with 500 µL of 0.05 M sodium acetate, then 110–200 MBq/mL ^68^GaCl_3_ eluted from the generator with 0.05 M HCl was added, and the pH of the final solution was adjusted to 4.0 − 4.5. After that, the reaction mixture of DOTA-RM2 was incubated for 10 min at 95℃. Finally, the AAZTA^5^-RM2 and DATA^5m^-RM2 were incubated for 5 min at room temperature.

In addition, we investigated the temperature and amount of precursor required for AAZTA^5^-RM2 and DATA^5m^-RM2 to complex with ^68^Ga^3+^. For temperature, all compounds were reacted at room temperature for 3, 5, and 15 min while other conditions remained the same. Specifically, 10 nmol of the precursor was added to 1 ml of ^68^GaCl_3_ (approximately 150 MBq) and the pH was adjusted using the same volume of sodium acetate buffer solution. For the amount of precursor, different amounts of precursor (~ 1 to 10 nmol) were added to the same volume of ^68^GaCl_3_ (150 MBq) and sodium acetate solution. DATA^5m^- and AAZTA^5^-were reacted for 10 min at room temperature and DOTA-for the same time at 95℃. Results were evaluated based on radiochemical yields.

Compounds were purified in order to investigate their biological effects accurately. First, the reaction mixture was diluted with 4 ml of water and purified through a pre-conditioned Oasis HLB cartridge (10 mg, Waters). The cartridge was then washed with 5 mL of deionized water. Finally, the product was eluted with EtOH (100 μL), and the radiochemical purity was analyzed with RP-HPLC.

### In vitro stability and Log *P*

The radioligand solutions were incubated in saline and human serum at 37℃ for 0.5–2 h to measure the in-vitro stability. For saline, a 20 µL solution can be extracted to monitor by RP-HPLC. For human serum, an equal volume of acetonitrile was added and mixed thoroughly, followed by centrifugation at 3000 rcf for 1 min. Then 20 µL supernatant was taken for RP-HPLC analysis.

The partition coefficient (log P) of each radiotracer was measured in octanol and PBS to measure the lipophilicity of the radioligands. Briefly, 20 μL of radiotracer in 480 μL of PBS was added to 500 μL of octanol in a centrifuge tube. The mixture was vigorously vortexed at room temperature for 2 min. After centrifuged for 1 min at 3000 rcf, 50 μL aliquots of both layers were measured using a gamma counter. The experiments were carried out in triplicate in three independent experiments.

### Cell-based

A GRPr-expressing human prostate cancer cell line PC-3 was cultured in F-12 K medium with 10% fetal bovine serum (FBS) and 1% penicillin/streptomycin. The cells were detached using a trypsin–EDTA solution. All experiments were performed in triplicate, and cells were seeded one days before the investigation.

Cellular uptake and internalization assay. PC-3 cells (1.5 × 10^5^ cells/well) were counted with a hemocytometer and were seeded in 24-well plates. The cells reached 80 to 90% confluence (3 × 10^5^ cells/well) in a day. Then the cells were washed twice with PBS, followed by adding 450 µL of F-12 K medium to each well. 50 µL of the corresponding radioligand were added to the medium and incubated (in triplicates) for 10, 30, 60 and 120 min at 37 °C, 5% CO_2_. To determine the uptake of the added radioactivity, the cells were washed three times with ice-cold PBS and lysed with 100 µL of 1 M NaOH. The internalized fraction was determined in the cells, washed with ice-cold PBS and then incubated for 10 min with acidic stripping buffer (0.05 M glycine stripping buffer in 100 mM NaCl, pH 2.8) followed by an additional washing step with ice-cold PBS and finally lysed with NaOH. All cell samples were measured with a γ-counter and presented as the percentage of added radioactivity.

In vitro binding specificity assay. PC-3 cells were incubated with ^68^Ga-X-RM2 solution for 30 min at 37 °C. One set of dishes in each experiment was pre-incubated with 20 µg unlabeled DOTA-RM2 added 30 min before the addition of the radiolabeled compounds in order to saturate the receptors. Then, the cells were washed three times with ice-cold PBS and lysed with NaOH. Cell associated radioactivity was measured in γ-counter.

### Imaging and biodistribution studies

Biodistribution. BALB/c nu/nu male mice were implanted subcutaneously with 10 million PC-3 tumor cells, freshly expanded in saline. Biodistribution studies were performed 3 to 4 weeks after tumor cell inoculation when the tumor-bearing mice reached an average mass of 20 ± 3 g. Mice were injected with ^68^Ga-X-RM2 (100 µL, 2.6 MBq) into the tail vein. The animals were sacrificed at 30, 60, and 120 min post-injection. Then the organs of interest were collected and weighed, and their radioactivity content was measured in a gamma counter.

Imaging. Tumor-bearing mice were injected with ^68^Ga-X-RM2 (100 µL, 2.6 MBq). All animals were placed in the prone position for micro-PET imaging at 30, 60, and 120 min post-injection. During the imaging process, mice were anaesthetized and maintained under 2% isoflurane in oxygen at a flow rate of 2 L/min. Images were recorded, reconstructed, and analyzed using Inveon-specific acquisition and research software packages. To examine the specificity of our compounds further, blocking studies were performed in the PC3 tumor-bearing nude mice. Briefly, the mice were injected with cold DOTA-RM2 (100 µg) in the tail vein, and 30 min later the ^68^Ga-X-RM2 was injected. Then, small animal PET/CT imaging was performed 30 min later to analyze the images and %ID/g values at the tumor site for comparison with the results of ^68^Ga-X-RM2 injection only.

### Statistical analysis

Quantitative analysis was performed using Student’s t-test using GraphPad Prism 9.0 software. Data are expressed as the mean ± standard deviation (SD). Statistical significance was defined at *p ≤ 0.05.

## Supplementary Information

**Additional file 1.** Additional information regarding the ESI-MS, biodistribution data, and HPLC analysis of the precursors is available through the supplementary information file.

## Data Availability

The datasets used and/or analyzed during the current study available from the corresponding author on reasonable request.

## Abbreviations

GRPr: The gastrin-releasing peptide receptor
BN: Bombesin
BB1/2/3/4: Bombesin receptor subtype 1/2/3/4
NMBR: Neuromedin B receptor
PSMA: Prostate-specific membrane antigen
PET: Positron emission tomography
PET/CT: Hybrid positron emission tomography with computer tomography
TOC: [DPhe^1^][Tyr^3^]-octreotide
FAP: Fibroblast activation protein
ESI-MS: Electrospray ionization-mass spectrometry
RP-HPLC: Reversed-phase high-performance liquid chromatography
PBS: Phosphate-buffered saline
FBS: Fetal bovine serum
SD: Standard deviation
