# Supplementary material for: The effects of novel macrocyclic chelates on the targeting properties of the 68Ga-labeled Gastrin releasing peptide receptor antagonist RM2
Source: EJNMMI Res. 2023 Jun 7;13:56. doi: 10.1186/s13550-023-01005-1 (PMC10247930; doi:10.1186/s13550-023-01005-1)

The effects of novel macrocyclic chelates on the targeting of the ^68^Ga-labeled Gastrin releasing peptide receptor antagonist RM2

Yinwen Wang^1,2,3^, Hongmei Yuan^1,2,3^, Sufan Tang^1,2,3^, Yang Liu^1,2,4^, Ping Cai^1,2,3^, Nan Liu^5^, Yue Chen^1,2,4,*^, Zhijun Zhou^1,2,3,4,^*

^1^The Department of Nuclear Medicine, Affiliated Hospital of Southwest Medical University, Jiangyang District, Luzhou, Sichuan, China

^2^Nuclear Medicine and Molecular Imaging Key Laboratory of Sichuan Province, Jiangyang District, Luzhou, Sichuan, China

^3^Department of Pharmaceutics, School of Pharmacy, Southwest Medical University, Jiangyang District, Luzhou, Sichuan, China

^4^Institute of Nuclear Medicine, Southwest Medical University, Jiangyang District, Luzhou, Sichuan, China

^5^Department of Nuclear Medicine, Sichuan Provincial People’s Hospital, University of Electronic Science and Technology of China, Chengdu, Sichuan, China

*Corresponding author: Zhijun Zhou, zhouzjiang@gmail.com, the Department of Nuclear Medicine, Affiliated Hospital of Southwest Medical University, Luzhou, China. Yue Chen, [chenyue5523@126.com](mailto:chenyue5523@126.com), the Department of Nuclear Medicine, Affiliated Hospital of Southwest Medical University.


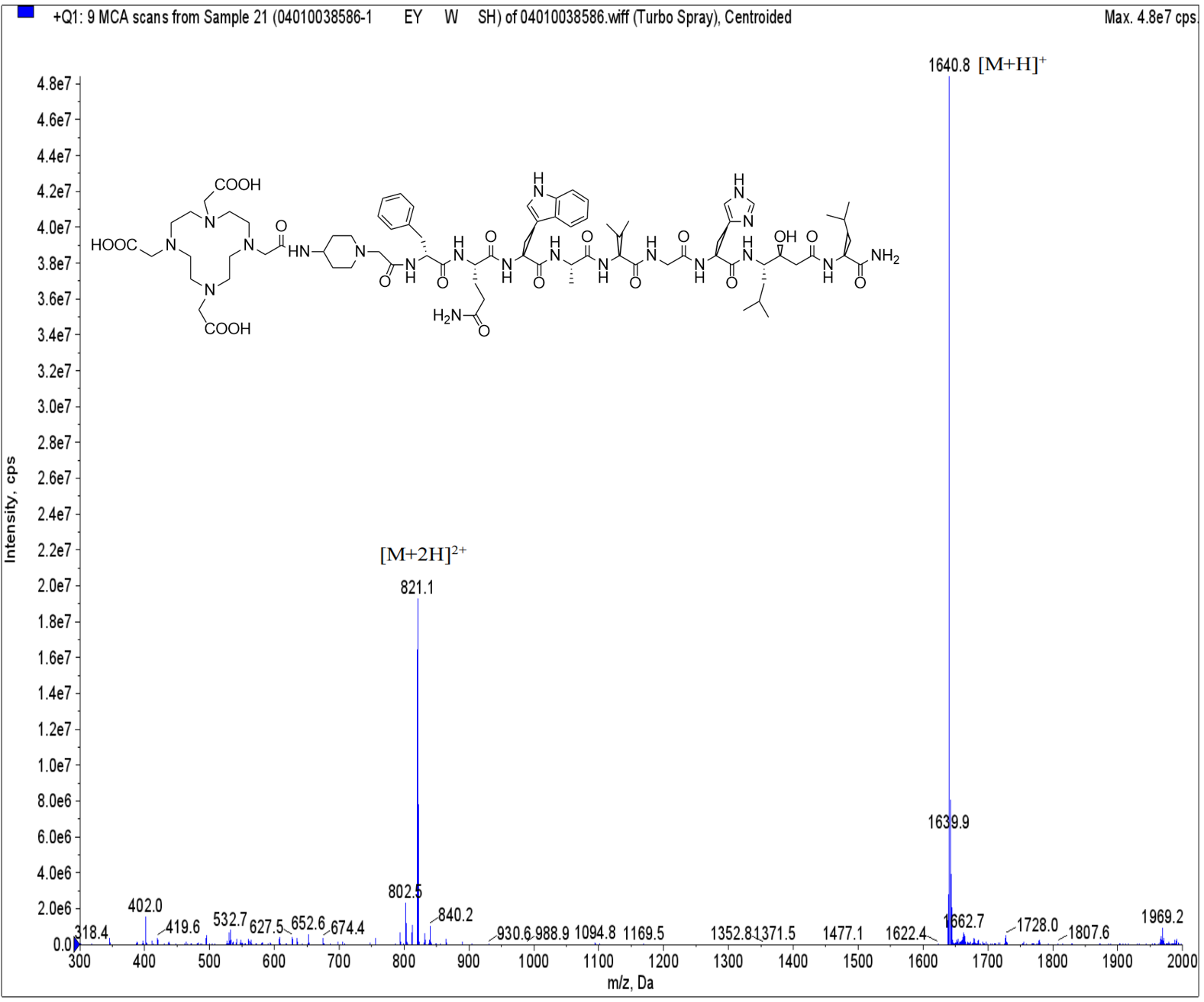


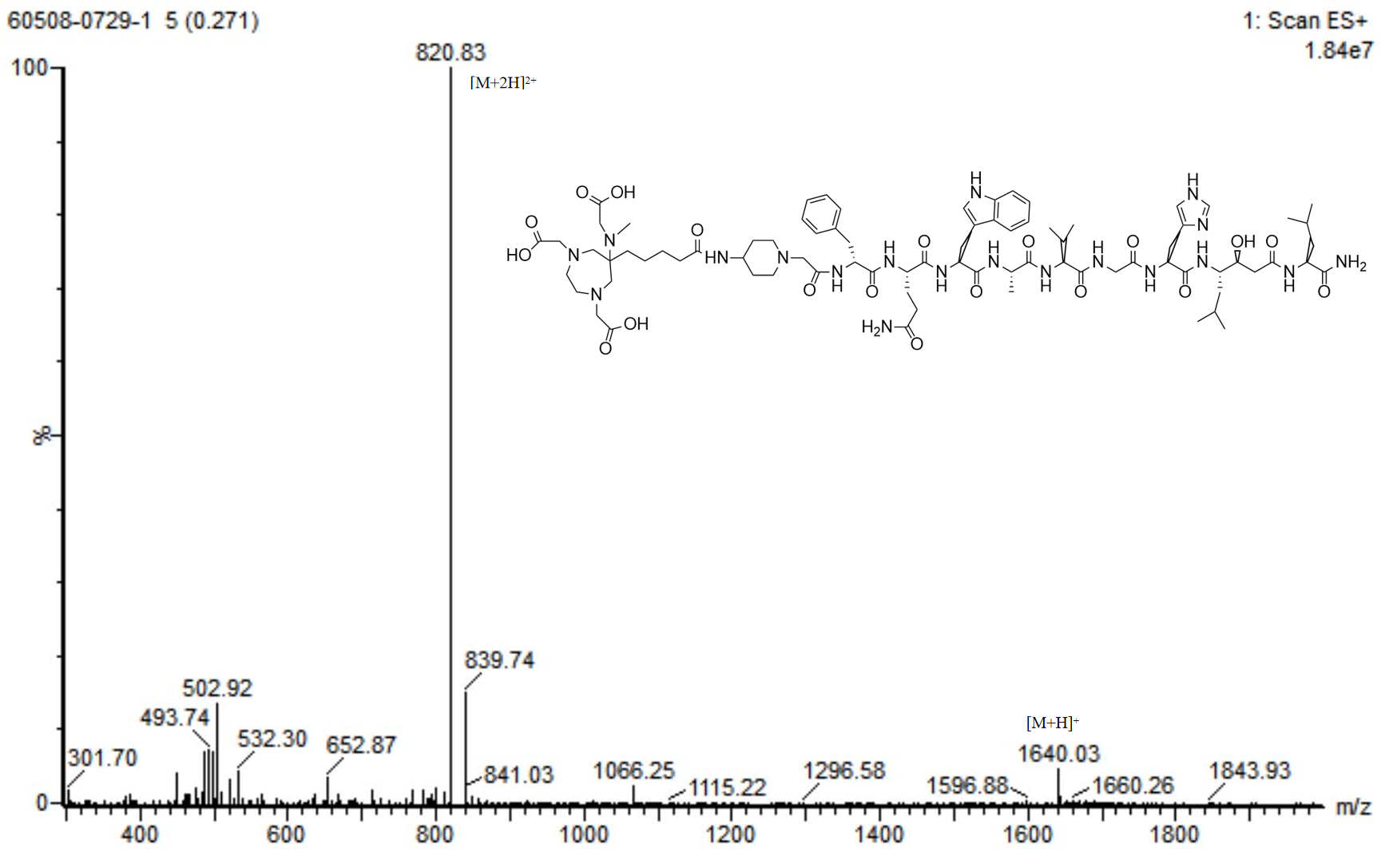


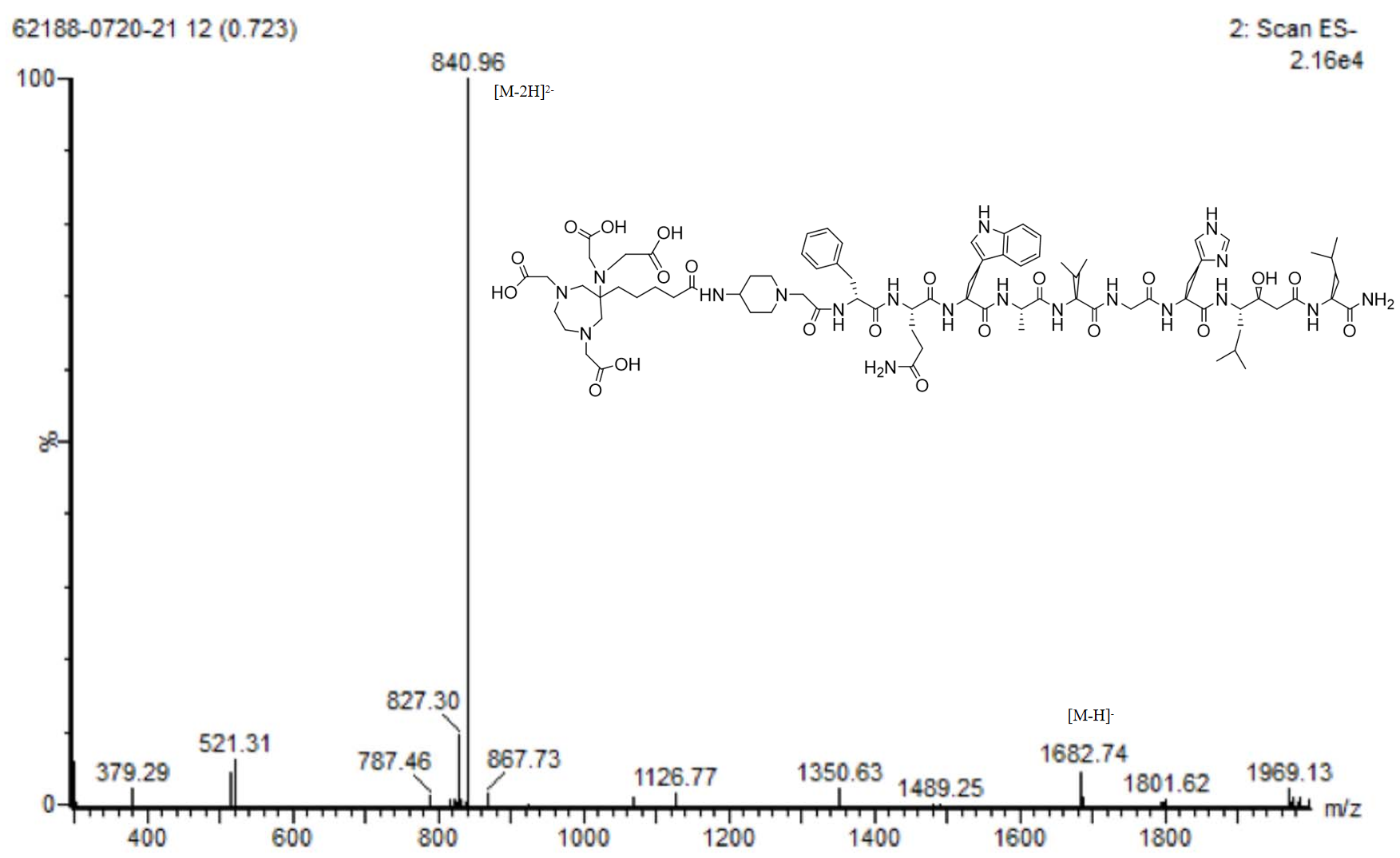


Figure S1. ESI-MS data for the X-RM2.

General Information:

The purity of all compounds was analyzed by using HPLC. HPLC condition: C18 Column: Agilent Eclipse Plus C18, 5 µm, 250 x 4.6 mm LC Column. For analytical runs, a linear gradient of solvent A (90–10% in 15 min) in solvent B at a flow rate of 1.0 mL/min was used for a 15 min run. Solvent A: acetonitrile mixed with 0.1% volume ratio of trifluoroacetic acid, Solvent B: ultrapure water mixed with 0.1% volume ratio of trifluoroacetic acid, and the wavelength of the ultraviolet detector is 220 nm.


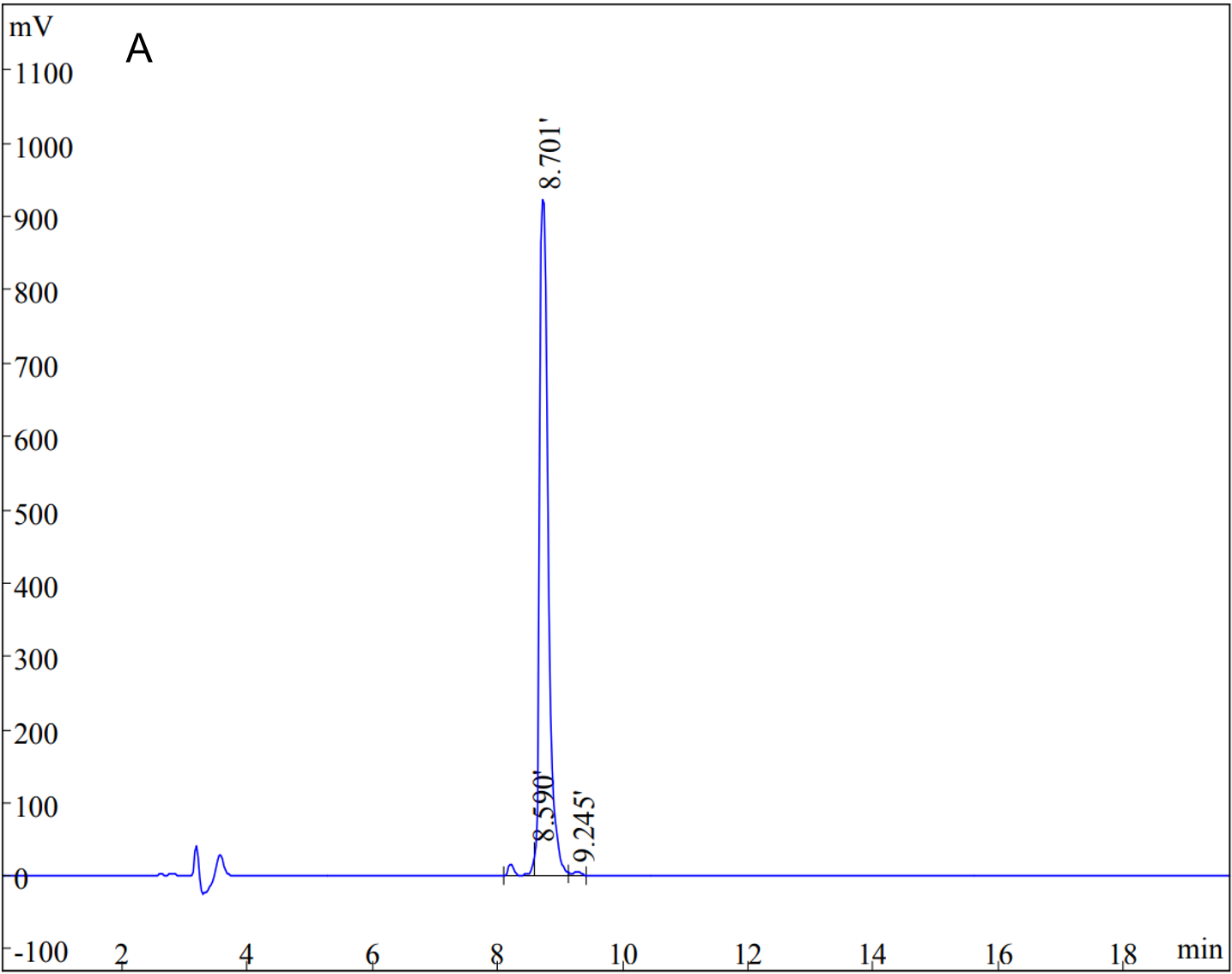


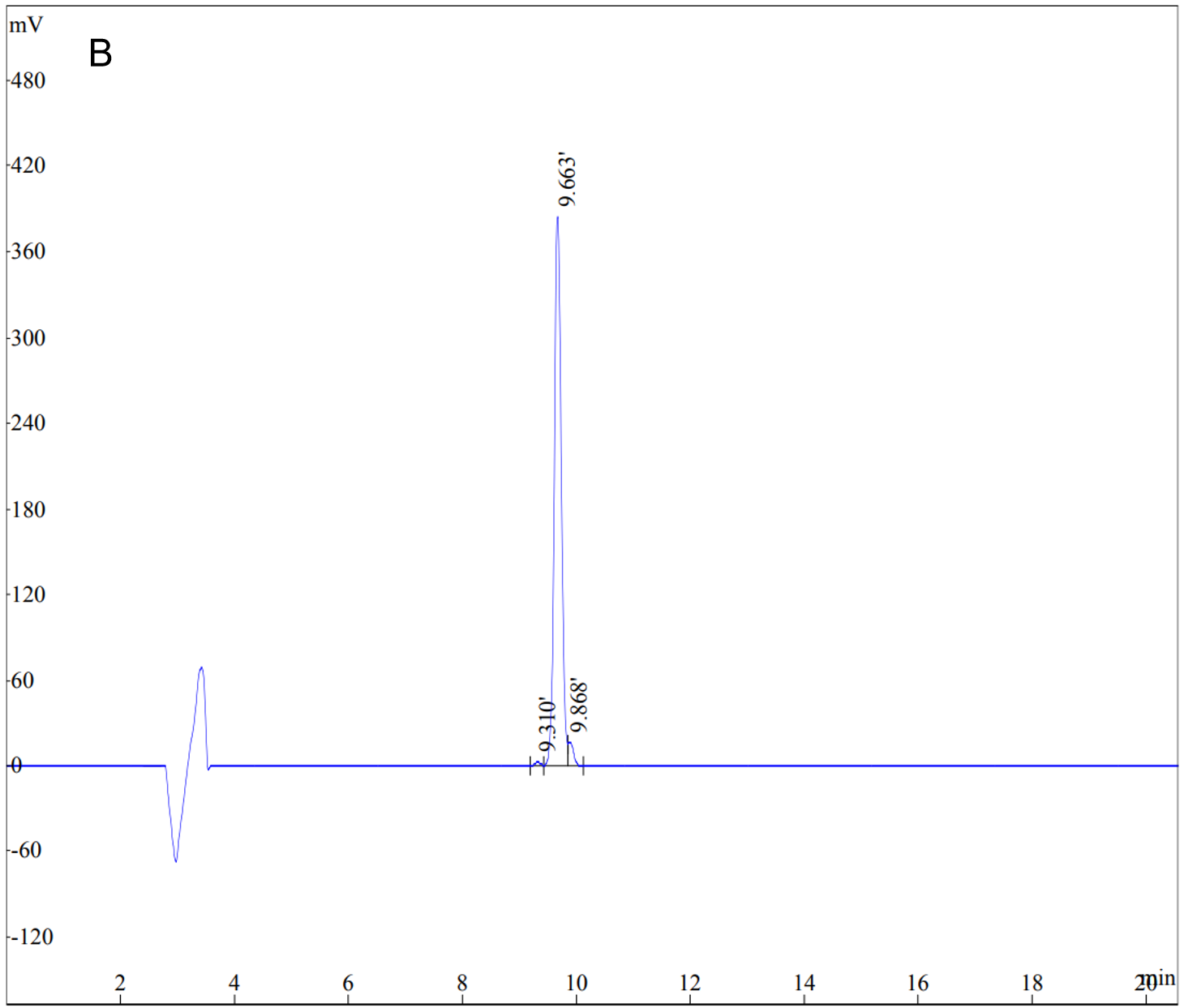


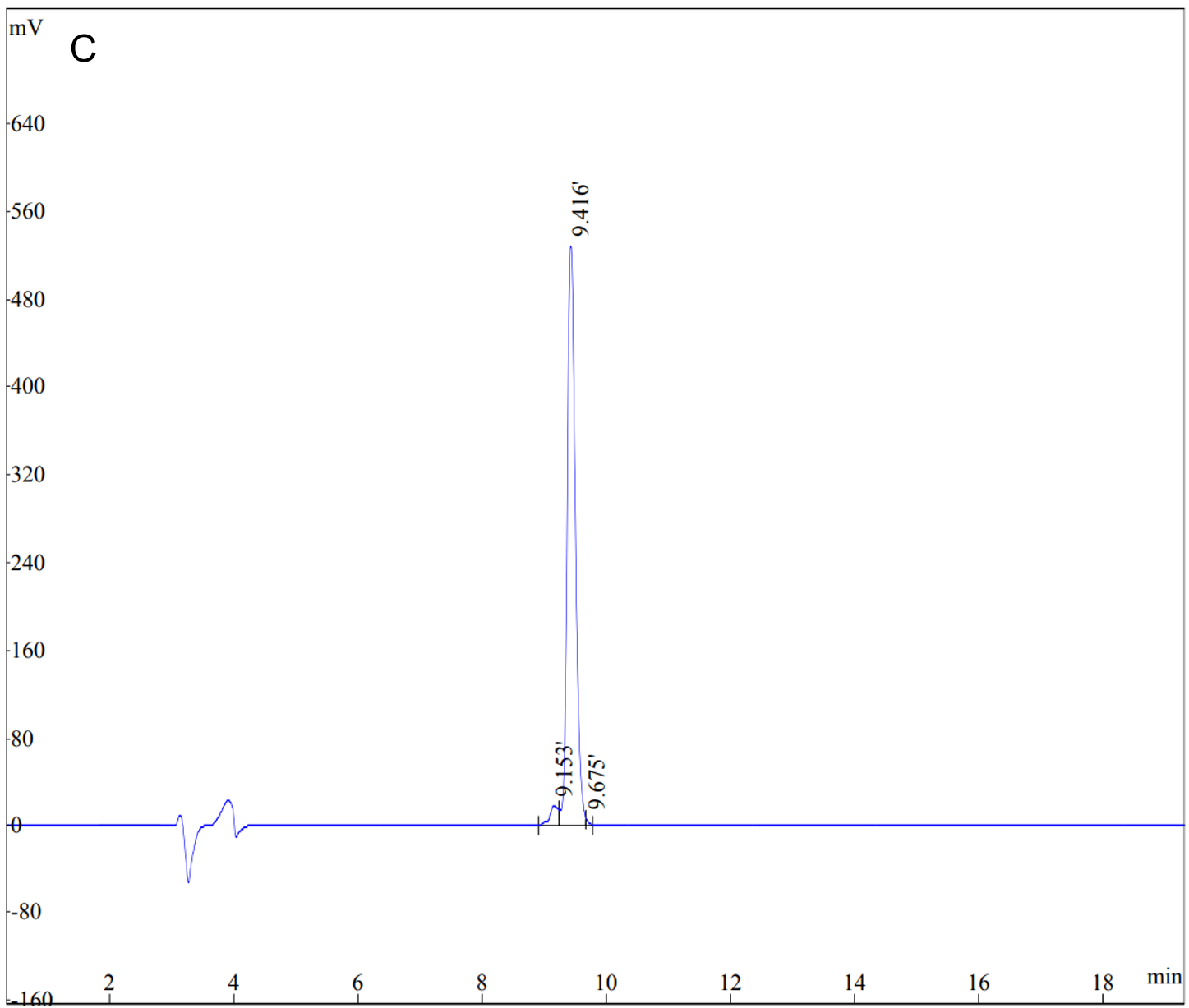


Figure S2. HPLC chromatograms of A) DOTA-RM2, B) DATA^5m^-RM2 and C) AAZTA^5^-RM2 at UV_220_.


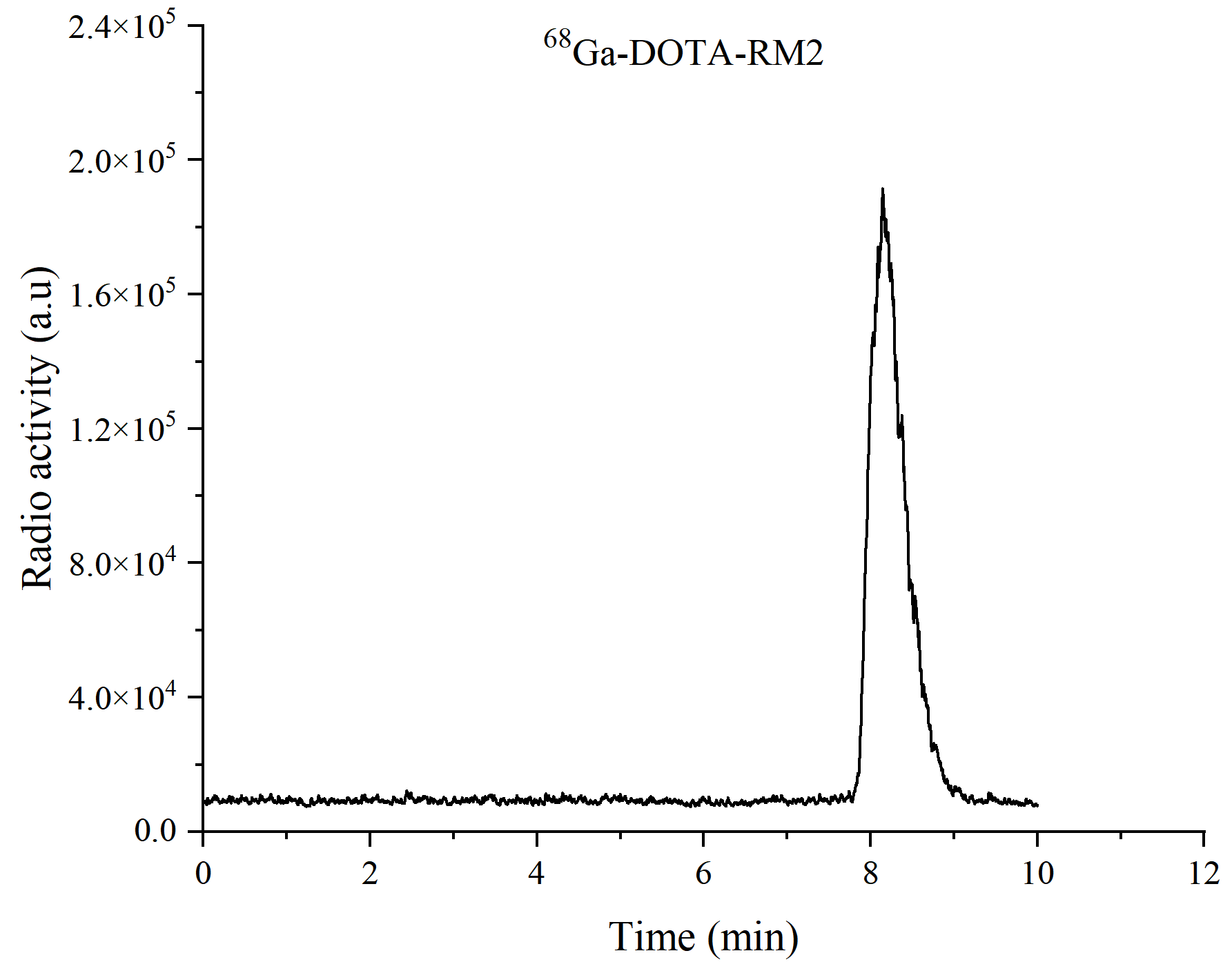

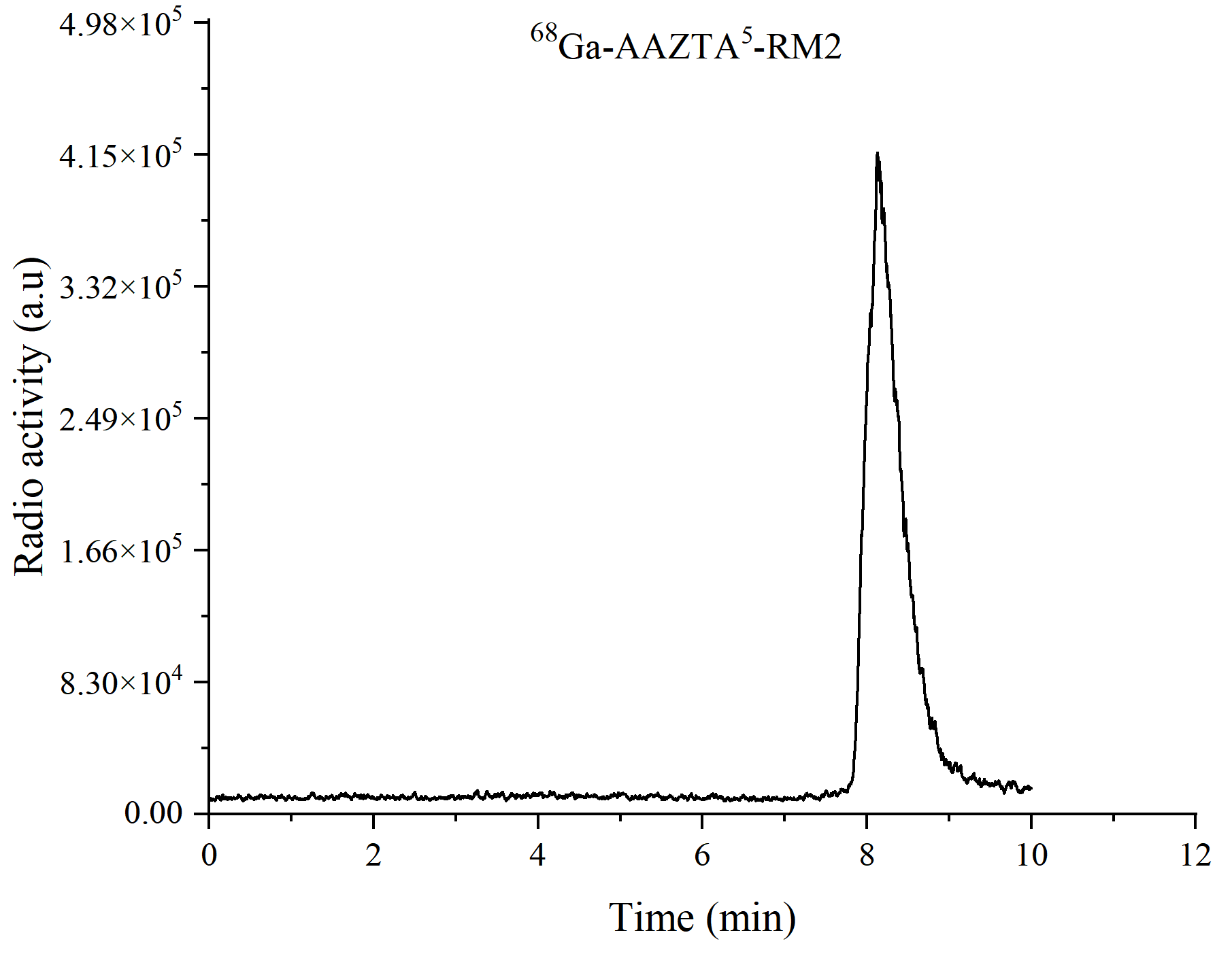


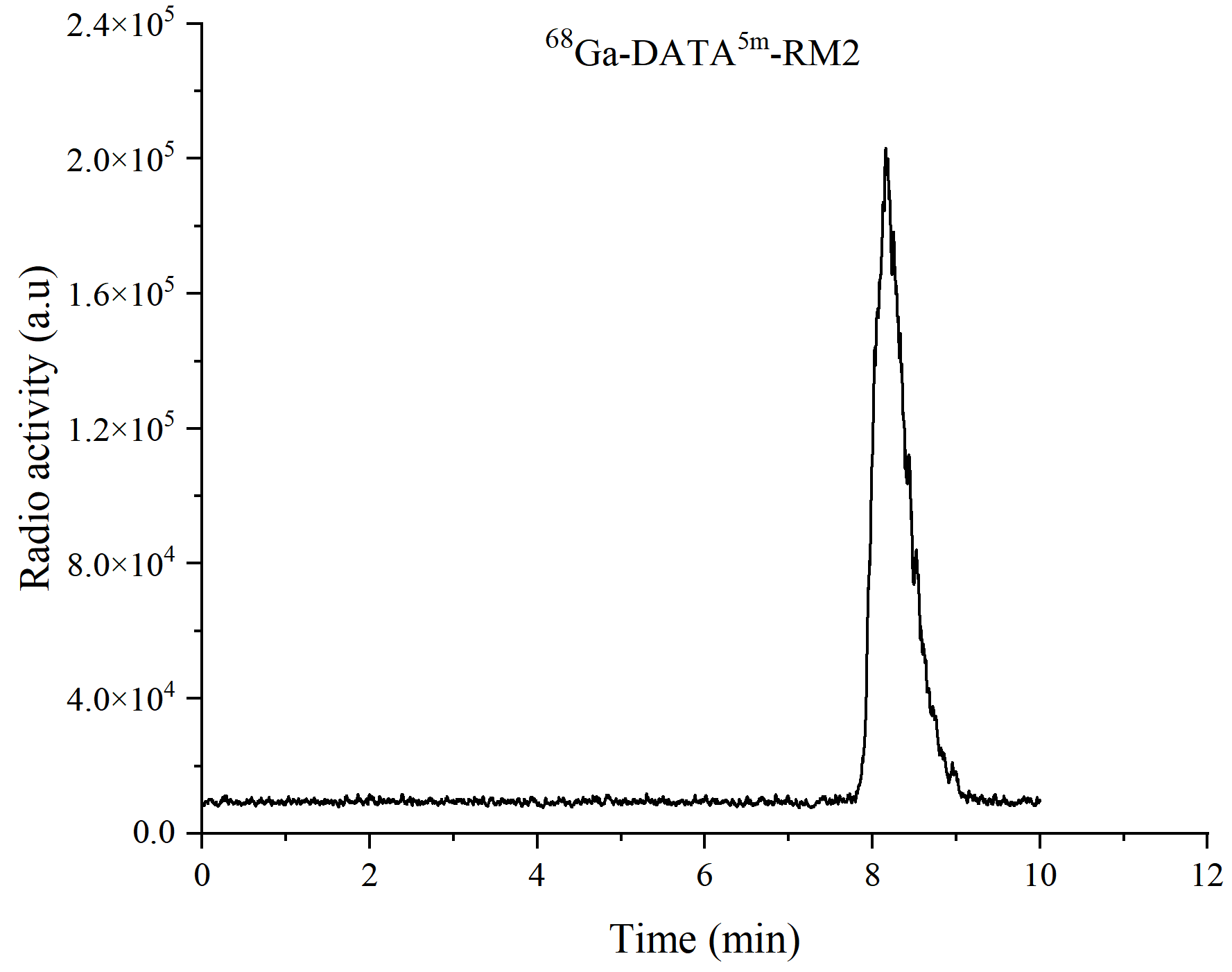


Figure S3. Radio-HPLC analysis of ^68^Ga-X-RM2.

Table S1. Biodistribution of ^68^Ga-X-RM2 in male nude mice bearing PC-3 tumours. Values are means ± SD %ID/g (n=5)


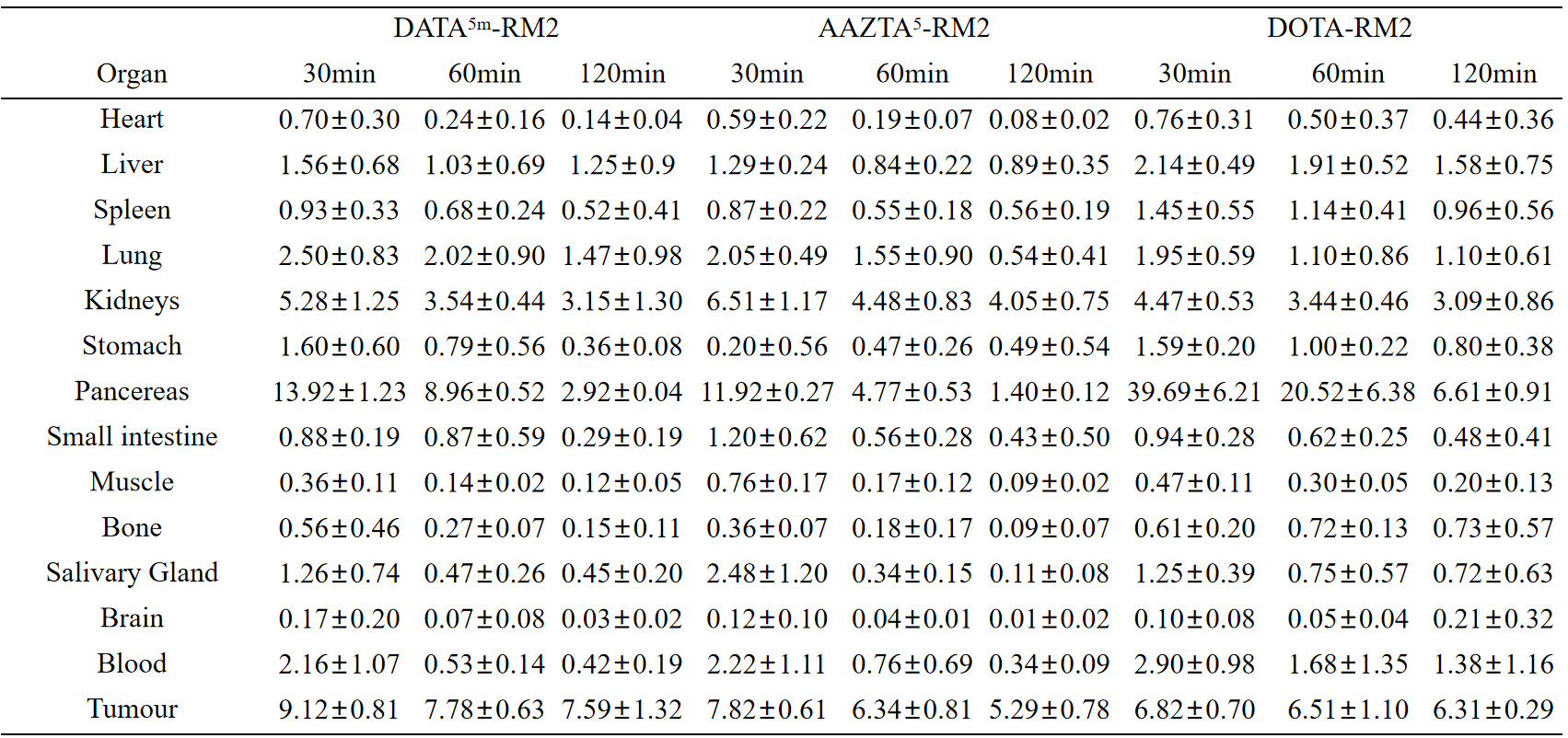

Supplement: Supplementary file 1 — Additional file 1. Additional information regarding the ESI-MS, biodistribution data, and HPLC analysis of the precursors is available through the supplementary information file. [file 13550_2023_1005_MOESM1_ESM.docx]
